# Impact of current and previous smoking on fracture risk in older women: the role of physical function, bone density, and bone microarchitecture

**DOI:** 10.1093/jbmr/zjag028

**Published:** 2026-02-10

**Authors:** Michail Zoulakis, Margarita Ambjörn, Raju Jaiswal, Kristian F Axelsson, Henrik Litsne, Lisa Johansson, Mattias Lorentzon

**Affiliations:** Sahlgrenska Osteoporosis Centre, Department of Internal Medicine and Clinical Nutrition, University of Gothenburg, 405 30 Gothenburg, Sweden; Department of Internal Medicine, Emergency Medicine and Geriatric Medicine, Sahlgrenska University Hospital, 431 80 Mölndal, Sweden; Sahlgrenska Osteoporosis Centre, Department of Internal Medicine and Clinical Nutrition, University of Gothenburg, 405 30 Gothenburg, Sweden; Sahlgrenska Osteoporosis Centre, Department of Internal Medicine and Clinical Nutrition, University of Gothenburg, 405 30 Gothenburg, Sweden; Sahlgrenska Osteoporosis Centre, Department of Internal Medicine and Clinical Nutrition, University of Gothenburg, 405 30 Gothenburg, Sweden; Region Västra Götaland, Närhälsan Norrmalm, Health Centre, 549 40 Skövde, Sweden; Sahlgrenska Osteoporosis Centre, Department of Internal Medicine and Clinical Nutrition, University of Gothenburg, 405 30 Gothenburg, Sweden; Sahlgrenska Osteoporosis Centre, Department of Internal Medicine and Clinical Nutrition, University of Gothenburg, 405 30 Gothenburg, Sweden; Department of Orthopedics, Sahlgrenska University Hospital, 431 80 Mölndal, Sweden; Sahlgrenska Osteoporosis Centre, Department of Internal Medicine and Clinical Nutrition, University of Gothenburg, 405 30 Gothenburg, Sweden; Department of Internal Medicine, Emergency Medicine and Geriatric Medicine, Sahlgrenska University Hospital, 431 80 Mölndal, Sweden

**Keywords:** osteoporosis, fracture, smoking, HR-pQCT, physical function, older women

## Abstract

While smoking is a known risk factor for fractures, its precise mechanisms among older women, particularly involving physical function, bone density, and bone microarchitecture, remain incompletely understood. This prospective cohort study included 3024 community-dwelling Swedish women aged 75-80 yr, followed for a median of 7.3 yr. Participants were categorized as current smokers (*n* = 157), former smokers (*n* = 1343), and never smokers (*n* = 1524). Radiographically verified fractures and all-cause mortality were assessed. Cox proportional hazards models, mediation analyses, and competing risk models evaluated associations between smoking status, fracture risk, and potential mediators, including walking speed and total volumetric BMD (vBMD). Current smokers had significantly increased risks of any fracture (HR 1.35; 95% CI 1.04-1.75) and hip fracture (HR 2.23, 95% CI 1.43-3.49) compared to never smokers. Former smokers exhibited intermediate risks. Women who had ceased smoking for 5-10 yr had substantially lower fracture risk than current smokers. Each year since cessation conferred an ~1% relative reduction in fracture and mortality risk. Mediation analyses revealed significant indirect effects via slower walking speed (18%-28%) and lower total vBMD, suggesting these factors are key contributors to fracture risk. Importantly, competing risk models confirmed elevated fracture risk in smokers even after accounting for increased mortality. These findings demonstrate that smoking is associated with increased fracture risk in older women, partly through impairments in physical function and vBMD. Smoking cessation appears to confer meaningful skeletal benefits, indicating a need for integrated strategies targeting both behavior change and physical function to reduce fracture burden in aging populations.

## Introduction

Osteoporotic fractures pose a substantial public health burden worldwide, with approximately 8.9 million such fractures occurring annually.^1^ These events often profoundly impact quality of life by precipitating prolonged disability, increased dependency, and elevated mortality rates, especially among the oldest segments of the population.^2^ Sweden ranks among the countries with the highest incidence of osteoporotic fractures, underscoring the urgency to identify modifiable risk factors and implement targeted prevention strategies.^3^

Tobacco smoking is recognized as a key contributor to bone loss and fracture risk^4^^,^^5^; however, the underlying pathways extend beyond direct skeletal effects.^6^ Biologically, cigarette smoke stimulates osteoclast activity, disrupts calcium and vitamin D metabolism, and alters hormonal balances critical for bone remodeling.^7–9^ Yet, clinical evidence increasingly suggests that smoking-related impairments in physical function, manifested by reduced muscle strength, slower walking speed, and diminished balance, also heighten fracture risk.^10^ Smokers often engage less in physical activity,^11^ thereby compounding bone fragility and predisposition to falls.^12^

Recent studies have deepened our understanding of these mechanisms. For instance, smoking increases pro-inflammatory cytokines, such as interleukin-6 (IL-6) and TNF-alpha (TNF-α), which enhance osteoclastogenesis and bone resorption.^13^ Moreover, oxidative stress induced by tobacco smoke has been shown to impair osteoblast function, leading to decreased bone formation.^14^ Advanced imaging techniques, such as High Resolution peripheral Quantitative Computed Tomography (HR-pQCT), have revealed that smokers exhibit significant deficits in both trabecular and cortical microarchitecture that standard bone mineral density (BMD) measurements might not fully capture.^15^

Despite growing evidence linking smoking to fracture risk, several uncertainties remain. For instance, it remains unclear whether these risks abate fully or partially when individuals quit smoking. Some research has shown that cessation can mitigate tobacco-induced bone turnover abnormalities,^16–18^ whereas other studies suggest persistent deficits in muscle performance^19^ or residual bone microarchitectural damage^20^ and increased fracture risk.^21^^,^^22^ Moreover, the precise contributions of physical function and skeletal deterioration as mediators of fracture risk remain incompletely delineated.

This study addresses these gaps by leveraging data from a large, population-based cohort of older Swedish women recruited into the Sahlgrenska University Hospital Prospective Evaluation of Risk of Bone fractures (SUPERB) study. This study aimed to examine the combined mediation roles of physical function and advanced bone-density measures, filling critical knowledge gaps. The objectives were 3-fold: first, to quantify fracture risk among current smokers compared to former and never smokers; second, to evaluate physical function and both an HR-pQCT-derived total vBMD and Dual X-ray Absorptiometry (DXA)-derived femoral neck (FN)BMD as potential mediators; and third, to explore the extent to which fracture risk decreases following smoking cessation. By clarifying the interplay of smoking, cessation, bone health and physical function, our findings could guide interventions aimed at reducing the considerable morbidity and mortality linked to osteoporotic fractures in aging populations.

To our knowledge, this is the first study to integrate smoking intensity and time since cessation with HR-pQCT microarchitecture derived bone characteristics and objective functional performance in a unified mediation framework for radiographically verified fractures, while accounting for competing mortality.

## Materials and methods

### Study design and subjects

The SUPERB cohort represents a population-based prospective study conducted in Gothenburg, Sweden, from 2013 to 2016. A total of 3028 women aged 75-80 yr were randomly identified through the National Population Register and contacted via letter or telephone. At the baseline examination, weight and height were measured using standardized equipment, and DXA as well as HR-pQCT of the distal radius and distal tibia were performed. Physical function tests were administered, and each participant completed a self-administered questionnaire detailing lifestyle factors, smoking and alcohol consumption habits, physical activity, medical history, medication use, and prior fractures. Prescription data were retrieved from the Swedish Prescribed Drug Register. All participants provided informed consent, and the study was approved by the Regional Ethics Review Board in Gothenburg. The examinations took place at the Clinical Osteoporosis Research Department, Sahlgrenska University Hospital in Mölndal, Sweden.

### Inclusion process

A total of 6832 women from the greater Gothenburg region, aged 75-80 yr, were invited to participate in SUPERB using information obtained from the Swedish National Registry. Eligibility criteria included the ability to walk with or without assistance, comprehension of the Swedish language, and having at least one hip that could be measured by DXA. Among the 6832 women initially contacted, 3368 (52.6%) declined or did not respond, and 436 met exclusion criteria, resulting in a final cohort of 3028 (47.4%) women. Another 4 women were excluded from this analysis because information on their smoking habits information was missing.

### Definition of smoking status groups

Participants were classified into 3 distinct groups based on their self-reported smoking history obtained from a validated questionnaire administered at baseline. Current smokers were defined as individuals actively smoking at the time of enrolment, for whom additional details regarding the age of initiation and daily cigarette consumption were recorded. Former smokers were those with a prior history of smoking who had subsequently quit, with the duration of cessation documented to allow further stratification by time since quitting. Participants reporting no history of tobacco use were categorized as never smokers.

### DXA and vertebral fracture assessment

DXA was used to measure BMD at the FN, lumbar spine (LS) from L1 to L4, and at one-third of the non-dominant radius. The trabecular bone score (TBS) of L1-L4 was determined using a Discovery A (Hologic). An additional 33 women were assessed using a Hologic QDR 4500/A Delphi DXA system. The cross-calibration between the 2 devices was conducted and has been described in detail in previous publications.^23^ LS BMD and TBS were calculated as the mean of at least 2 assessable vertebrae in the L1-L4 region, excluding any vertebrae that were fractured or contained osteosynthesis material. The coefficient of variation (CV) for FN-BMD was 1.3%, for LS-BMD was 0.7%, and for TBS was 2.12%. Vertebral fractures were identified using lateral DXA scans and graded semi-quantitatively per Genant.^24^ Vertebral fracture assessment (VFA) was conducted by 2 experienced physicians, as previously described.^25^

### High-resolution peripheral quantitative computed tomography

Measurements of the distal radius and distal tibia on the non-dominant side were performed with an HR-pQCT device (XtremeCT; Scanco Medical AG). For the distal radius, the first CT slice was positioned 9.5 mm proximal to the articular surface, and for the distal tibia, it was placed 22.5 mm proximal to the articular surface. A more proximal region (at 14% of the bone length from the articular surface) was used as it is represented by a relatively larger proportion of cortical bone. Each scan site generated 110 cross-sectional images, which were graded according to image quality. Only images with a quality score between 1 and 3 were used in the analyses, as recommended by the manufacturer (Scanco Medical AG). Parameters obtained included trabecular bone volume fraction (BV/TV, %), trabecular number (mm^−1^), trabecular thickness (mm), trabecular separation (mm), cortical volumetric BMD (Ct.vBMD, mg/cm^3^), cortical area (mm^2^), and total volumetric BMD (mg/cm^3^). To determine CVs, duplicate measurements were performed on 30 older women. The CVs for trabecular parameters ranged from 0.4% to 2.5% in the distal radius and from 0.8% to 2.6% in the distal tibia. For cortical parameters in the distal radius and distal tibia, the CVs ranged from 0.1% to 0.9%. Using the Image Processing Language (IPL v5.08b) software provided by Scanco Medical AG, the cortical bone was isolated from both trabecular bone and extraosseous soft tissue through automatically placed contours along periosteal and endosteal surfaces, with manual corrections made when necessary. From these images, cortical pore volume (Ct.Po.V; mm^3^), cortical bone volume (Ct.BV; mm^3^), and cortical porosity (Ct.Po; %) were derived, where cortical porosity was calculated by Ct.Po.V/(Ct.Po.V + Ct.BV). The CVs for cortical porosity measurements ranged from 5.3% to 13.3% for the distal radius and from 0.9% to 4.1% for the distal tibia.

### Bone microindentation

Bone material strength index (BMSi) was assessed using the OsteoProbe device, by performing microindentations at the midshaft of the tibia after applying local anesthesia. The midshaft was defined as the midpoint between the distal apex of the patella and the medial malleolus. At least 11 indentations were made in a circular pattern, with the first indentation discarded to ensure that the probe was properly positioned on the cortical surface. Only valid indentations, a minimum of 10 per participant, were used for analysis. The OsteoProbe software then graded the stability of the indentations as unstable, stable, or very stable. Five operators conducted the procedure and to ensure consistency, at least 2 operators were present during the first 100 measurements. The intra-observer CV was 3.2%, and the inter-observer CV was 5.2%, as previously described.^26^ A total of 642 women underwent microindentation using this method.

### Anthropometrics and questionnaires

Body height and weight were recorded using standardized equipment, specifically the same wall-mounted calibrated stadiometer and scale for all individuals. The mean values of 2 consecutive measurements were used to determine height and weight. Data regarding age, medical history, medication use, family history of fractures, prior fractures, alcohol consumption, self-reported falls in the past 12 mo, and smoking status were gathered through a validated self-administered questionnaire. The questionnaire also addressed alcohol consumption, defining excessive intake as 3 or more alcoholic beverages per day. The 12-item Physical Component Summary (PCS-12) for physical health and its mental health counterpart, the Mental Component Summary (MCS-12), were included.^27^ The Charlson comorbidity index was employed to assess comorbidity.^28^ Data on medical conditions were collected at baseline from the questionnaires, supplemented by information from hospital and outpatient specialized clinics using ICD-10 codes obtained from the National Patient Register.

### Physical function tests

Walking speed was determined by instructing participants to walk 10 m at a preferred pace.^29^ The timing began after the first 2 m and ended at 8 m, thereby excluding acceleration and deceleration. The mean of 2 such trials was recorded in meters per second. The timed up and go (TUG) test was administered to assess mobility and balance by measuring the time (in seconds) required for participants to rise from a chair, walk 3 m at a casual pace, turn around, return to the chair, and sit down.^30^^,^^31^ Walking aids were permitted as necessary. Balance was evaluated using the one leg standing (OLS) test,^32^^,^^33^ which measured how long the participant could stand on one leg (up to a maximum of 30 s) with eyes open and arms folded across the chest. This test was performed twice for both legs, and the best value was used in the analyses. Lower body strength was measured by the 30-s chair-stand test, during which participants rose from a chair (without armrests and with arms crossed over the chest) and sat back down repeatedly for 30 s, with the total number of stands recorded.^34^ Grip strength of the dominant hand was measured using a Saehan hydraulic hand dynamometer (model SH5001; Saehan Corporation) as previously described.^35^ With the elbow flexed at a 90° angle and the forearm resting on a table, participants performed 2 attempts, and the average force in kilograms was recorded. The Physical Activity Scale for the Elderly (PASE)^36^ questionnaire was used to estimate physical activity in the week preceding enrollment.

### Blood analyses

Blood samples were obtained at baseline, and both plasma and serum were promptly stored at −80 °C until analysis. Serum albumin and creatinine were assayed at the Department of Clinical Chemistry (accredited testing laboratory, Swedac no. 1342), Linköping University Hospital, Sweden, with reagents from the same batch and run on a cobas c 701 instrument (Roche Diagnostics Scandinavia AB). Serum albumin was measured immunoturbidimetrically (range: 3.0-101 g/L, total CV ≤4% at 2 different albumin concentrations). Serum creatinine was measured enzymatically (range: 5-2700 μmol/L, total CV ≤5% at 2 different creatinine concentrations). Estimated glomerular filtration rate (eGFR) was calculated using the Lund–Malmö revised equation. Hemoglobin was analyzed at the Department of Clinical Chemistry (accredited testing laboratory, Swedac no. 1240), Sahlgrenska University Hospital, Gothenburg, Sweden, using a CN-free hemoglobin method on the ADVIA 2120i system (Siemens Healthcare GmbH), with an analytical range of 0-22.5 g/dL and total CVs <1.5% at 3 distinct hemoglobin levels. Serum 25OHD levels were determined on a DiaSorin LIAISON XL analyzer, which exhibits 100% cross-reactivity for both 25-hydroxyvitamin D2 and D3 and has an analytical range of 10-375 nmol/L with total CVs of 8.8%, 6.4%, and 6.8% at 25, 68, and 150 nmol/L, respectively. Serum intact PTH was measured using an Elecsys immunoassay on a Roche Cobas e601 platform, with an analytical range of 0.13-530 pmol/L and total CVs of 4.0% and 2.9% at 1.9 and 8.6 pmol/L, respectively.

### Incident fractures and mortality

Incident fractures were ascertained through a regional X-ray archive encompassing 49 municipalities in the Västra Götaland region surrounding Gothenburg, Sweden. All reported fractures were verified by 5 research nurses who examined radiology reports from baseline (March 2013 to April 2016) until the end of March 2023. Radiographs lacking radiology reports or with uncertain fracture diagnoses were manually evaluated by an experienced orthopedic surgeon. The mortality data were sourced from the regional population registry, Västfolket.

### Statistical methods

Comparisons of baseline characteristics across the 3 smoking categories (current, former, and never smokers) were performed using one-way analysis of variance (ANOVA) with post hoc least significant difference (LSD) tests for continuous variables, and either chi-squared or Fisher’s exact tests for categorical variables. Fracture outcomes were defined as any fracture, major osteoporotic fracture (MOF), and hip fracture. The associations between smoking status and fracture incidence were analyzed using Cox proportional hazards models with stepwise adjustment: (1) age and BMI; (2) FRAX clinical risk factors (previous fragility fracture, parental hip fracture, excessive alcohol consumption, glucocorticoid use, rheumatoid arthritis, and secondary osteoporosis); and (3) FN-BMD. In sensitivity analyses, we further adjusted for comorbidity and treatment variables (Charlson comorbidity index, estrogen therapy, osteoporosis medications [bisphosphonates, denosumab, strontium ranelate, and PTH analogues], daily calcium supplementation, and calcium intake from dairy; “Model 3 + Other”) and for fall/balance proxies (self-reported falls in the prior 12 mo and One-Leg Standing [OLS] time; “Model 3 + falls and OLS”). Measures of functional performance (walking speed) were added as specified.

For the sensitivity analysis, smoking status was further categorized into 3 groups: never-smoker (reference), low (1-10 cigarettes/day), and moderate and heavy (11+ cigarettes/day). An alternative stratification considered current smokers as the reference group, with ex-smokers classified by years since cessation (0-5, 5-10, 10-15, and 15 yr).

Missing data on clinical risk factors (CRFs) included in FRAX were addressed using multivariate imputation by chained equations (MICE) in RStudio (version 1.4.1106, Posit), with 20 iterations applied to 208 participants (6.9% of the cohort) as previously described.^37^^,^^38^ All regression models were implemented in SPSS (version 29, IBM) and R, with analyses conducted between September 2024 and February 2025.

To explore how smoking was associated with fracture risk, mediation analyses were conducted using the R mediation package. Separate mediator models for walking speed, FN-BMD, and total vBMD were regressed on smoking status, adjusting for confounders, such as age, BMI, and CRFs. The total effect of smoking on fracture risk was divided into the average causal mediated effect (ACME) and the average direct effect (ADE), with quasi-Bayesian CIs computed from 1000 simulations to determine the proportion mediated by each factor. Linear regressions assessed the association between smoking status and each mediator, while logistic regressions related smoking status, the mediator, and the binary outcome. The mediate function from the R package provided ACME, ADE, and the proportion mediated via a nonparametric bootstrap approach (1000 simulations), with the Sobel test used for additional assessment of the indirect effect. Variance inflation factors (VIF) were <1.30, indicating no substantial multicollinearity. All predictor and mediator variables were centered to facilitate interpretation and mitigate multicollinearity. In sensitivity analyses, the framework was repeated with TUG and 30-s Chair-Stand test as alternative functional mediators.

Given the advanced age of the studied population, competing risk analyses were conducted to account for mortality as an event that could preclude fracture. The Aalen–Johansen estimator was employed to derive cumulative incidence functions (CIFs), and Gray’s test was used to compare CIFs by smoking status. Additionally, Fine and Gray subdistribution hazard models were applied to estimate SHRs for fractures, treating death as a competing event. All models reported HRs or SHRs with 95% CIs, and *p*-values below .05 were considered statistically significant.

## Results

### Baseline characteristics

At baseline, the cohort comprised 5.2% current smokers, 44.4% former smokers, and 50.4% never smokers. Statistical analysis revealed no clinically significant differences in age across the groups, although minor variations were present. Current smokers were characterized by a slightly lower body height, weight, and BMI, alongside a higher total body lean mass compared to never smokers. Additionally, smokers reported reduced self-assessed physical activity and poorer mental health (Table 1).

**Table 1:** Baseline characteristics.

	**Current smokers** ***N* = 157**	**Former smoker** ***N* = 1323**	**Never smoker** ***N* = 1524**	***p***
Age (yr)	77.7 ± 1.6	77.7 ± 1.6^*^	77.9 ± 1.6^*^	.005
Weight (kg)	64.6 ± 11.9^*^	69.3 ± 12.6^*^^*^	68.8 ± 11.6^*^,^*^^*^	<.001
Height (cm)	161.0 ± 52.7^*^	162.1 ± 5.7^*^,^*^^*^	161.7 ± 6.1^*^	.03
BMI (kg/m^2^)	24.9 ± 4.3^*^,^*^^*^	26.3 ± 4.6^*^^*^	26.3 ± 4.3^*^	<.001
Appendicular lean mass (kg/m^2^)	6.3 ± 0.8^*^,^*^^*^	6.0 ± 0.8^*^^*^	6.3 ± 0.8^*^	<.001
PCS-12^a^	44.3 ± 10.4	44.5 ± 11.4^*^	45.8 ± 10.5^*^	.003
MSC-12^a^	51.0 ± 11.2^*^,^*^^*^	53.3 ± 9.6^*^	54.0 ± 8.9^*^^*^	<.001
Previous fracture	46 (29.3%)	498 (37.1%)	571 (37.5%)	.13
Family history of fracture	17 (18.3%)	246 (18.3%)	268 (17.6%)	.06
Oral glucocorticoid exposure	6 (3.8%)	37 (2.8%)	60 (3.9%)	.21
Rheumatoid arthritis	9 (5.7%)	52 (3.9%)	59 (3.9%)	.51
Alcohol (3 or more units/day)^b^	1 (0.6%)^*^	16 (1.2%)^*^^*^	0 (0.0%)^*^,^*^^*^	.001
Vertebral fracture on VFA^c^	36 (23.7%)	318 (24.6%)	351 (23.8%)	.86
Secondary osteoporosis^d^	36 (22.9%)	250 (18.6%)	293 (19.2%)	.43
Dietary calcium intake mg	320 (389)	375 (359)	378 (368)	.17
Smoking duration^e^^,^^f^ (yr)	59.5 ± 5.86	27.0 ± 26.0	-	<.001
Pack years	27.58 ± 15.33	14.79 ± 13.44	-	<.001
Average cigarettes per day^e^	10 ± 6	10 ± 7	-	.04
Charlson comorbidity index (mean)	1.10 ± 1.42	1.04 ± 1.54	0.93 ± 1.47	.35
Charlson comorbidity index (score)				.055^e^
0	76 (48.4%)	662 (49.3%)	824 (54.1%)	
1	31 (19.7%)	279 (20.8%)	323 (21.2%)	
2	27 (17.2%)	244 (18.2%)	235 (15.4%)	
3	11 (7.0%)	95 (7.1%)	79 (5.2%)	
4 or more	12 (7.6%)	63 (4.7%)	63 (4.7%)	
FRAX hip without FN BMD^g^	21.5 ± 17.0^*^,^*^^*^	14.9 ± 13.4^*^^*^	15.1 ± 13.1^*^	<.001
FRAX hip with FN BMD^g^	12.0 ± 10.9^*^,^*^^*^	7.0 ± 9.0^*^^*^	7.0 ± 8.5^*^	<.001
FRAX MOF without FN BMD^g^	32.9 ± 18.0^*^,^*^^*^	32.5 ± 16.0^*^^*^	30.5 ± 16.6^*^	.002
FRAX MOF with FN BMD^g^	22.7 ± 14.2^*^,^*^^*^	19.5 ± 13.6^*^^*^	20.0 ± 13.2^*^^*^	.006
**Blood biochemistry**				
Calcium (mmol/L)	2.48 ± 0.10	2.46 ± 0.09	2.47 ± 0.10	.25
25-Hydroxyvitamin D_3_ (nmol(L)	57.9 ± 21.0^*^,^*^^*^	62.2 ± 21.5^*^^*^	62.9 ± 21.0^*^	.02
0-25 (nmol/L)	9 (5.7%)	31(2.3%)	27 (1.8%)	.006
0-50 (nmol/L)	55 (35.0%)	392 (29.2%)	416 (27.3%)	.10
PTH (pmol/L)	4.85 ± 1.9	5.18 ± 2.4^*^	4.95 ± 2.1^*^	.011
Phosphate (mmol/L)	1.23 ± 0.16	1.19 ± 0.15	1.18 ± 0.14	<.001
Albumin (g/L)	42.8 ± 2.8	42.7 ± 2.9	42.9 ± 2.8	.49
Creatinine (μmol/L)	72.27 ± 14.0^*^,^*^^*^	78.25 ± 19.9^*^^*^	77.54 ± 18.1^*^	<.001
Hemoglobin (g/L)^h^	138 ± 11.7^*^,^*^^*^	135.4 ± 10.8^*^^*^	135.1 ± 10.7^*^	.001
EGFR^d^ (mL/min/1.73 kvm)	75.60 ± 20.63^*^	69.73 ± 20.98	69.49 ± 20.59^*^	<.001
**CKD stadium**				.06
CKD 1-2	129 (82.2%)	997 (74.2%)	1130 (74.1%)	
CKD 3	28 (17.8%)	329 (24.5%)	384 (25.2%)	
CKD 4-5	0 (0.0%)	17 (1.3%)	10 (0.7%)	

a N Former smokers = 1341, Never smokers = 1341.

b Fisher’s exact test.

c N Smokers = 152, N Former smokers = 1291, N Never smokers = 1477.

d Diabetes mellitus, chronic liver disease, inflammatory bowel disorder, untreated hyperthyroidism, or premature menopause.

e Median ± IQR and Mann–Whitney U test.

f N Smokers = 153, Former smokers = 1185.

g Median ± IQR and Kruskal–Wallis test with Bonferroni adjustment for group-to-group comparisons.

h N Smokers = 155, N Former smokers = 1318, Never smokers = 1497.

Cohort characteristics are presented as mean ± SD. Group differences were investigated using ANOVA with least significant difference (LSD) comparison. For categorical variables numbers and percent within each group are presented. The differences in proportions of categorical variables were tested using Chi squared tests. Significant values are shown in bold.

^*^/^*^^*^/^*^^*^^*^ showing significance between pair groups.

Abbreviation: VFA, vertebral fracture assessment.

Despite these differences, there were no significant disparities in clinical risk factors, such as previous fractures, family history of fractures, glucocorticoid use, rheumatoid arthritis, self-reported falls, secondary osteoporosis, or comorbidity burden as measured by the Charlson comorbidity index. The prevalence of vertebral fractures detected via VFA using DXA did not differ significantly among the groups (Table 1).

In terms of blood analysis, calcium levels were comparable across all smoking statuses with no statistically significant differences. However, vitamin D levels were significantly lower in current smokers compared to former and never smokers. Similarly, smokers exhibited lower serum PTH and serum creatinine levels relative to the other groups. Conversely, smokers had slightly elevated hemoglobin levels that were statistically significant when compared to both former and never smokers. Serum albumin levels remained consistent across all groups without significant variations (Table 1).

Baseline FRAX scores for both MOF and hip fractures were higher in smokers compared to former and never smokers, both with and without adjustment for FN-BMD, indicating a higher predicted 10-yr fracture risk in the smoking population (Table 1).

### Bone parameters and physical function

Bone health assessments demonstrated that smokers had lower FN-BMD, total hip BMD, and TBS compared to former and never smokers. Lumbar spine BMD was marginally lower in smokers, while at the ultra-distal tibia site, smokers had reduced cortical volumetric BMD (vBMD) and cortical area (Table 2). Additionally, total vBMD and the trabecular bone volume fraction (BV/TV) were lowest among smokers, with significant differences observed between groups. Trabecular separation (Tr.Sp) was elevated in smokers, and stiffness and failure load were markedly lower compared to former and never smokers. The cortical perimeter of the radius showed no significant differences, although cortical porosity was slightly increased in smokers relative to never smokers. In contrast, bone microindentation derived BMSi did not differ significantly between groups (Table 2).

**Table 2:** Bone characteristics and physical function tests and sarcopenia.

	**Smoker**	**Former smoker**	**Never smoker**	***p***
**Bone characteristics**				
Femoral neck BMD g/cm^2^	0.64 ± 0.11^*,^^*^^*^	0.66 ± 0.1^*^	0.66 ± 0.10^*,^^*^^*^	.01
BMD total Hip g/cm^2^	0.77 ± 0.13^*,^^*^^*^	0.80 ± 0.12^*^	0.80 ± 0.12^*^^*^	<.001
BMD LS g/cm^2^	0.94 ± 0.17	0.95 ± 0.17^*^^*^	0.94 ± 0.17^*^	.04
BMD radius 1/3 g/cm^2^	0.57 ± 0.08	0.58 ± 0.08	0.58 ± 0.08	.08
Trabecular bone score	1.19 ± 0.12^*,^^*^^*^	1.21 ± 0.11^*,^^*^^*^^*^	1.21 ± 0.11^*^^*,^^*^^*^^*^	.003
BMSi	79.0 ± 8.1	78.3 ± 7.4	77.8 ± 7.3	.71
**Ultra distal tibia microarchitecture**				
Total area (mm^2^)	728.2 ± 103.9	733.1 ± 105.0	726.1 ± 106.1	.22
Cortical vBMD (mg/cm^3^)	720.2 ± 77.1^*,^^*^^*^	736 ± 68.6^*,^^*^^*^^*^	743.6 ± 68.1^*^^*,^^*^^*^^*^	<.001
Cortical area (mm^2^)	70.1 ± 25.9^*,^^*^^*^	77.5 ± 23.0^*,^^*^^*^^*^	79.5 ± 26.0^*^^*,^^*^^*^^*^	<.001
Total vBMD (mg/cm^3^)	206.5 ± 49.8^*,^^*^^*^	224.7 ± 47.6^*^	228.9 ± 48.0^*^^*^	<.001
Trabecular thickness (mm)	0.065 ± 0.013^*,^^*^^*^	0.069 ± 0.013^*^	0.069 ± 0.013^*^^*^	.002
Trabecular separation (mm)	0.54 ± 0.16^*,^^*^^*^	0.52 ± 0.13^*^	0.51 ± 0.13^*^^*^	.011
Trabecular BV/TV (%)	0.11 ± 0.033^*,^^*^^*^	0.12 ± 0.029^*^	0.12 ± 0.030^*^^*^	<.001
Cortical perimeter (mm)	105.7 ± 7.60	106.2 ± 7.70	105.7 ± 7.80	.19
Cortical porosity (%)	12.5 ± 4.0	12.5 ± 3.9^*^	12.0 ± 4.0^*^	.003
Stiffness (kN/mm)	149.4 ± 31.7^*,^^*^^*^	164.0 ± 28.5^*^	164.1 ± 28.6^*^^*^	<.001
Failure load (N)	7643 ± 1565^*,^^*^^*^	8350 ± 1382^*^	8349 ± 1395^*^^*^	<.001
**Ultra distal radius microarchitecture**				
Total area (mm^2^)	271.5 ± 44.0	272.4 ± 43.5	269.0 ± 45.6	.18
Cortical vBMD (mg/cm^3^)	766.1 ± 82.0	765.7 ± 77.8^*^	773.8 ± 80.0^*^	.04
Cortical area (mm^2^)	36.9 ± 11.9	37.3 ± 11.5	37.7 ± 11.6	.55
Total vBMD (mg/cm^3^)	233.5 ± 63.9	239.2 ± 61.0	242.0 ± 63.9	.31
Trabecular thickness (mm)	0.0584 ± 0.013	0.0583 ± 0.011	0.0579 ± 0.011	.62
Trabecular separation (mm)	0.63 ± 0.29^*,^^*^^*^	0.57 ± 0.23^*^	0.58 ± 0.22^*^^*^	.03
Trabecular BV/TV (%)	9.6 ± 3.6	10.2 ± 3.4	10.0 ± 3.4	.17
Cortical perimeter (mm)	69.6 ± 5.7	70.2 ± 5.9	69.7 ± 6.2	.12
Cortical porosity (%)	4.7 ± 2.1	4.6 ± 2.4^*^	4.3 ± 2.0^*^	<.001
Stiffness (kN/mm)	53.8 ± 13.0	55.2 ± 12.2	54.4 ± 12.2	.24
Failure load (N)	2755 ± 653	2827 ± 607	2781 ± 603	.15
**Physical function and sarcopenia**				
Walking speed (m/s)	1.16 ± 0.25^*,^^*^^*^	1.25 ± 0.26^*,^^*^^*^^*^	1.28 ± 0.24^*^^*,^^*^^*^^*^	<.001
30s Chair stand (n)	9.5 ± 4.4^*,^^*^^*^	10.5 ± 4.6^*^	10.7 ± 4.2^*^^*^	.005
Grip strength (kg)	14.6 ± 5.4	15.0 ± 5.5	14.5 ± 5.5	.06
One leg stand (s)	12.3 ± 9.6	14.0 ± 9.5	14.0 ± 9.7	.18
PASE score	75.7 ± 57.4^*,^^*^^*^	96.8 ± 69.3^*^^*^	99.2 ± 67.6^*^	<.001
Time up and go (s)	8.7 ± 2.8^*,^^*^^*^	8.0 ± 2.7^*^^*^	7.9 ± 2.5^*^	<.001
Self-reported falls	46 (29.3%)	424 (31.6%)	425 (27.9%)	.10
AWGS	31 (20.3%)	126 (9.5%)	152 (9.5%)	<.001
EWGSOP2	35 (22.4%)	164 (12.3%)	177 (11.7%)	<.001
SDOC	9 (5.8%)	71 (5.3%)	54 (3.6%)	.053

Cohort characteristics are presented as mean ± SD with least significant difference (LSD) comparison. Group differences were investigated using ANOVA with LSD comparison.

^*^/^*^^*^/^*^^*^^*^ showing significance between groups.

Abbreviations: AWGS, Sarcopenia according to Asian Working Groups for sarcopenia criteria; PASE, Physical Activity Scale for the Elderly; EWGSOP2, Sarcopenia according to European Working Group on Sarcopenia in Older People criteria; SDOC, Sarcopenia according to Sarcopenia Definitions and Outcomes Consortium.

Detailed numbers of individuals with available data for each variable are provided in Table S4.

Regarding physical function and sarcopenia parameters, smokers performed worse on 3 out of 5 physical function tests and reported lower scores on the PASE questionnaire. Specifically, smokers had 7.8% and 10.3% slower walking speeds, performed 10.5% and 12.6% fewer chair stands, and took 8.0% and 9.2% longer time to complete the TUG test compared to former and never smokers, respectively. A significantly higher proportion of smokers met the criteria for sarcopenia according to the Asia Working Group for Sarcopenia (AWGS) and the European Working Group on Sarcopenia in Older People (EWGSOP2), though marginally non-significant differences were noted based on the Sarcopenia Definitions and Outcome Consortium (SDOC) criteria, compared to nonsmokers (Table 2).

### The risk of fracture and death

Over a median follow-up of 7.3 yr, a total of 1082 fractures were recorded, including 238 hip fractures. Compared to never smokers, current smokers exhibited a 35% increased risk of any fracture (HR, 1.35; 95% CI, 1.04-1.75; *p* = .03) and a 36% elevated risk of MOF (HR, 1.36; 95% CI, 1.00-1.83; *p* = .05). The risk of hip fractures was particularly pronounced, with current smokers facing more than double the risk (HR, 2.23; 95% CI, 1.43-3.49; *p* < .001) (Table 3).

**Table 3:** Hazard ratios for fractures and mortality.

**Fracture risk**	**Any HR (95% CI)**	***p***	**MOF HR (95% CI)**	***p***	**Hip HR (95% CI)**	***p***	**Death HR (95% CI)**	***p***
Never smoker	1 (Reference)		1 (Reference)		1 (Reference)		1 (Reference)	
No. (%)	516 (33.9%)		388 (25.5%)		114 (7.5%)		235 (15.4%)	
								
**Smoker**								
No. (%)	65 (41.4%)		49 (31.2%)		24 (15.3%)		57 (36.3%)	
Unadjusted	1.39 (1.07-1.80)	.01	1.40 (1.04-1.88)	.03	2.34 (1.51-3.64)	<.001	2.58 (1.93-3.44)	<.001
Model 1	1.39 (1.08-1.80)	.01	1.41 (1.04-1.89)	.03	2.25 (1.45-3.51)	<.001	2.74 (2.05-3.66)	<.001
Model 2	1.46 (1.13-1.90)	.004	1.49 (1.10-2.00)	.01	2.42 (1.55-3.77)	<.001	2.69 (2.01-3.61)	<.001
Model 3	1.37 (1.06-1.80)	.02	1.38 (1.02-1.87)	.04	2.27 (1.45-3.55)	<.001	2.69 (2.01-3.61)	<.001
Model 3 + Other^a^	1.46 (1.13-1.90)	.004	1.48 (1.10-2.00)	.01	2.35 (1.50-3.67)	<.001	2.57 (1.92-3.45)	<.001
Model 3 + Walking Speed^b^	1.24 (1.00-1.62)	.11	1.26 (0.94-1.70)	.14	2.01 (1.28-3.17)	.003	2.06 (1.52-2.79)	<.001
Model 3 + Falls and OLS^c^	1.34 (0.98-1.83)	.07	1.50 (1.05-2.13)	.03	2.07 (1.20-3.55)	.009	2.17 (1.45-3.24)	<.001
								
**Former smoker**								
No (%)	501 (37.3%)		371 (27.6%)		100 (7.4%)		274 (20.4%)	
Unadjusted	1.12 (1.01-1.30)	.04	1.12 (0.97-1.29)	.12	1.02 (0.78-1.34)	.88	1.36 (1.14-1.62)	<.001
Model 1	1.15 (1.01-1.30)	.03	1.13 (0.98-1.30)	.10	1.04 (0.79-1.36)	.79	1.39 (1.17-1.65)	<.001
Model 2	1.16 (1.02-1.31)	.02	1.14 (0.99-1.32)	.07	1.04 (0.80-1.36)	.76	1.40 (1.17-1.66)	<.001
Model 3	1.16 (1.02-1.31)	.02	1.13 (0.98-1.31)	.09	1.04 (0.80-1.37)	.77	1.40 (1.17-1.67)	<.001
Model 3 + Other^a^	1.15 (1.01-1.30)	.03	1.14 (0.98-1.31)	.08	1.03 (0.78-1.35)	.83	1.42 (1.19-1.69)	<.001
Model 3 + Walking Speed^b^	1.23 (0.97-1.25)	.11	1.10 (0.94-1.26)	.28	0.98 (0.74-1.29)	.87	1.29 (1.03-1.14)	.005
Model 3 + Falls and OLS^c^	1.18 (1.02-1.36)	.02	1.20 (1.02-1.42)	.03	1.06 (0.76-1.46)	.75	1.54 (1.24-1.92)	<.001

a All covariates used in model 3 with the addition of Charlson Comorbidity index, daily calcium supplementation, osteoporosis treatment (bisphosphonates, denosumab, strontium, and PTH), hormonal replacement therapy (estrogen) and calcium intake from dairy.

b All covariates used in model 3 with the addition of mean walking speed.

c All covariates used in model 3 with the addition of self-reported falls (12 mo before inclusion) and One Leg Standing (OLS) test.

Hazard ratios (HR) and 95% CI from Cox regression models. Significant values are shown in bold.

Model 1: adjusted for age and BMI.

Model 2: all covariates used in model 1 with the addition of Clinical risk factors included in FRAX: previous fractures after 50 yr of age, secondary osteoporosis, rheumatoid arthritis, oral glucocorticoid use with at least 5 mg daily and for 3 mo or more, parental history of hip fracture, excessive alcohol intake (3 units per day or more).

Model 3: all covariates used in model 2 with the addition of FN BMD T-score.

Former smokers demonstrated intermediate fracture risks, highlighting the potential benefits of smoking cessation. These associations were attenuated upon inclusion of walking speed in the models (Table 3). Adjustments for falls and OLS produced a modest additional attenuation for hip fracture, but did not substantially affect associations with any fracture and MOF. In contrast, inclusion of calcium supplementation, osteoporosis therapies, and estrogen therapy (Model 3 + Other) yielded small increases in fracture hazard ratios. Similarly, current and former smokers exhibited a higher mortality risk (HR, 2.60; 95% CI, 1.94-3.48; *p* < .001) and (HR, 1.40; 95% CI, 1.17-1.67; *p* < .001), respectively.

**Figure 1:**
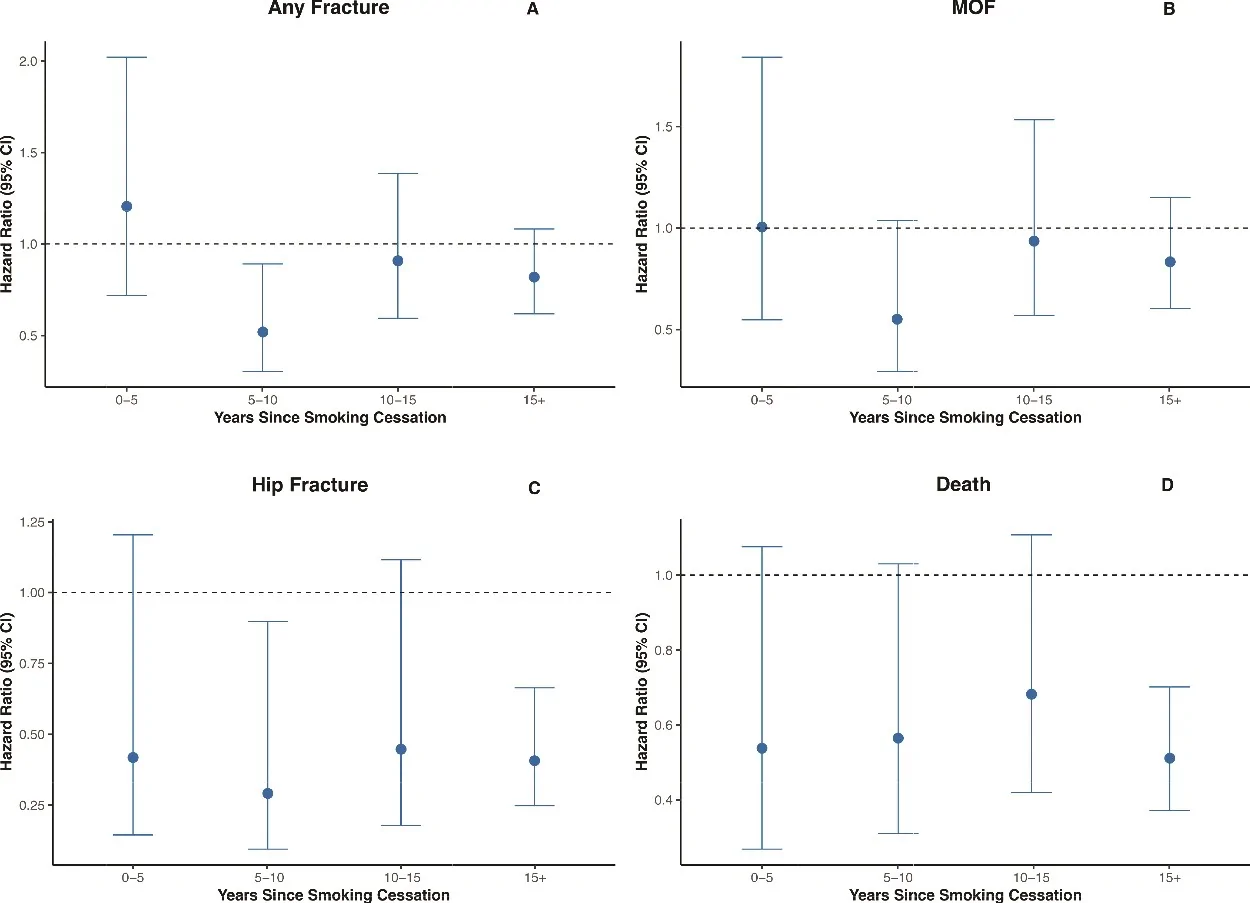
Hazard ratios (HRs) for fracture and mortality by smoking cessation duration. Hazard ratios with 95% CIs for any fracture (A), major osteoporotic fracture (MOF, B), hip fracture (C), and death (D), comparing ex-smokers to current smokers (reference group). Hazard ratios were estimated using Cox proportional hazards models, adjusted for pack-years, age, BMI, clinical risk factors (CRFs), and FN-BMD. The dashed line at HR = 1.0 represents the risk in current smokers.

Sensitivity analysis revealed differential associations between smoking cessation duration and the risks of fractures and mortality. Specifically, individuals who ceased smoking for 5-10 yr exhibited a statistically significant reduction in the risk for any fracture (HR, 0.52; 95% CI, 0.30-0.89; *p* = .02) and hip fracture at both 5-10 yr (HR, 0.29; 95% CI, 0.09-0.90; *p* = .03) and 15+ yr (HR, 0.41; 95% CI, 0.25-0.66; *p* < .001) compared to current smokers (Figure 1 and Table S1). Additionally, mortality risk was significantly lower for individuals who had ceased smoking for 15 or more years (HR, 0.51; 95% CI, 0.37-0.72; *p* < .001). In similarly adjusted Cox models, each year of smoking cessation was associated with a reduced risk of any fracture (HR, 0.99; 95% CI, 0.98-1.00; *p* < .01), hip fracture (HR, 0.99; 95% CI, 0.98-1.00; *p* = .02) and mortality (HR, 0.99; 95% CI, 0.99-0.99; *p* < .001), but not for MOF (HR, 1.00; 95% CI, 0.99-1.00; *p* = .13).

**Figure 2:**
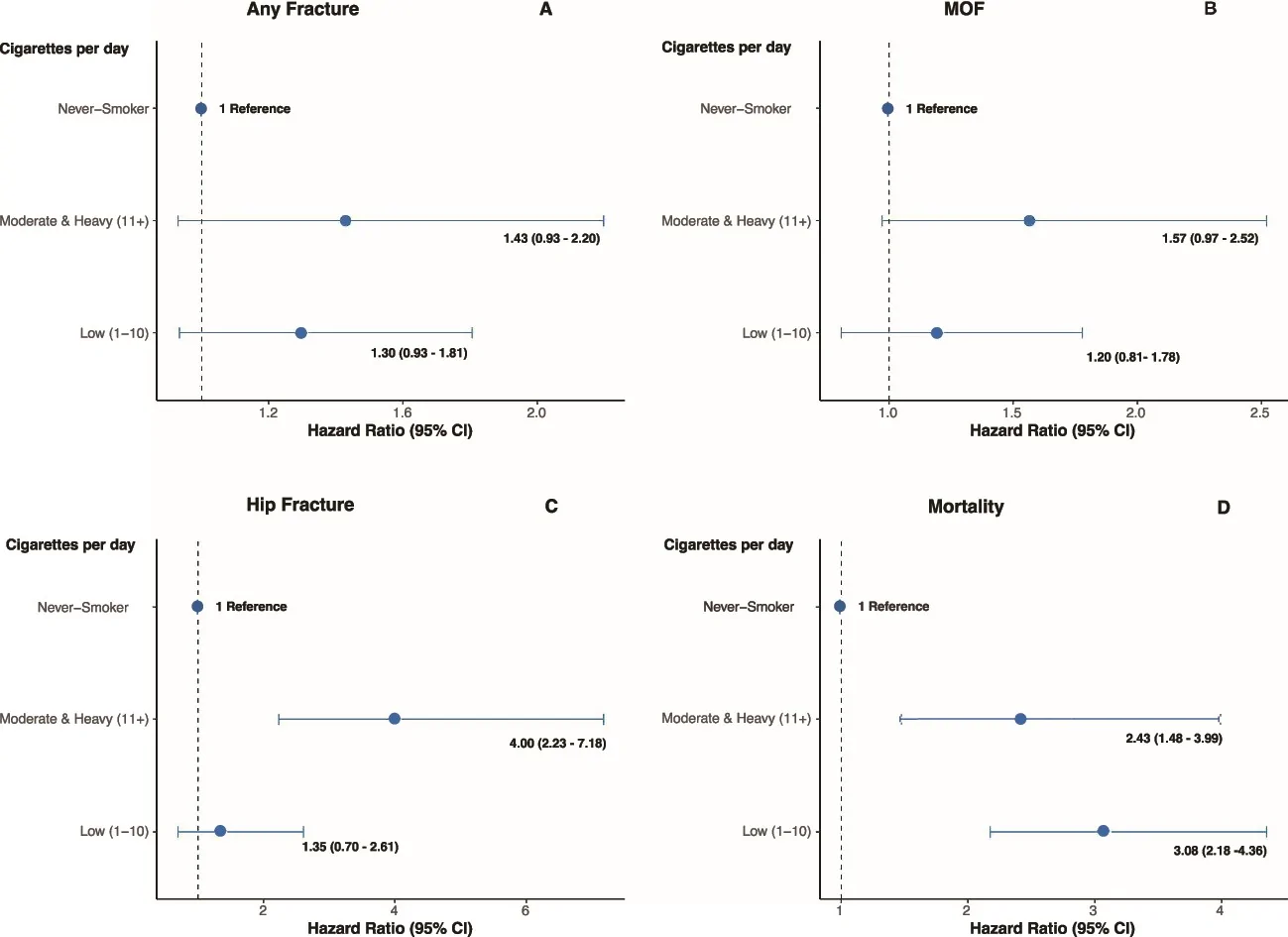
Hazard ratios for fracture and mortality by cigarette smoking exposure. The figure presents hazard ratios (HRs) with 95% CIs for any fracture (A), major osteoporotic fracture (MOF, B), hip fracture (C), and mortality (D) by cigarette smoking exposure. HRs were estimated using Cox proportional hazards models, adjusted for age, BMI, pack-years, clinical risk factors and FN-BMD. The dashed reference line at HR = 1.0 indicates no difference compared to the reference group.

In terms of smoking exposure, moderate and heavy current smokers demonstrated an elevated risk of hip fracture (HR, 4.00; 95% CI, 2.23-7.18; *p* < .001) and mortality (HR, 2.43; 95% CI, 1.48-3.99; *p* < .001) compared to never-smokers (Figure 2). Low-exposure smokers did not show statistically significant differences in fracture risks compared to never-smokers (Table S2). Each unit increase of cigarettes per day was associated with a 3% increase in mortality risk (HR, 1.03; 95% CI, 1.01-1.04; *p* < .001) and a 1% increase in the risk of both any fracture (HR, 1.01; 95% CI, 1.00-1.02; *p* = .01) and MOF (HR, 1.01; 95% CI, 1.00-1.02; *p* = .03). For hip fracture, this association was not significant (HR, 1.01; 95% CI, 0.99-1.03; *p* = .22).

### Mediation analyses

Across the 3 fracture outcomes: any fracture, hip fracture, and MOF mediation analyses were performed using three continuous mediators: walking speed, total vBMD, and FN-BMD. For walking speed, the average causal mediation effect (ACME) was statistically significant across all outcomes (eg, ACME ≈ 0.0034 for MOF, with similar magnitudes for any and hip fracture, *p* < .001). Similarly, total vBMD exhibited significant mediation effects (ACME ≈ 0.0038, *p* < .01) across outcomes. In contrast, although the ACME for FN-BMD reached significance in some analyses (eg, ACME ≈ 0.0016), the effect was not consistently supported by the Sobel tests (z ≈ 1.16, *p* > .05). Notably, the ADEs and total effects were not statistically significant in any of the models, implying that the overall effect of smoking status on fracture risk may be largely transmitted indirectly through these mediators. The proportion mediated estimates ranged from approximately 18%-28% for walking speed and total vBMD, whereas the estimates for FN-BMD were lower and non-significant (Table S3A).

In sensitivity mediation analyses replacing walking speed with TUG and 30-s Chair-Stand, TUG showed small but statistically significant indirect effects for any fracture (ACME ≈ 0.002; *p* = .008) and hip fracture (ACME ≈ 0.0005; *p* = .003), with a significant proportion mediated for any fracture (≈0.11; *p* = .04), whereas effects for MOF were directionally similar but not significant (*p* ≈ 0.11). Chair-Stand test was directionally consistent but not significant across outcomes (ACME *p* ≈ 0.15). Average direct effects were small and generally non-significant in these models, and total effects remained modest on the probability scale (Table S3B).

### Competing risks of death

For MOF, the 9-yr cumulative incidence was 32.9% (95% CI, 24.6-41.1) in current smokers compared to 27.8% (95% CI: 25.2-30.3) in never smokers. Although Gray’s test did not reveal a statistically significant difference (*p* = .181), the Fine and Gray model indicated a trend toward increased MOF risk among current smokers (SHR = 1.36; 95% CI, 0.998-1.85; *p* = .051) (Tables S4 and S5 and Figure S1).

Hip fractures demonstrated a more pronounced disparity, with a 9-yr cumulative incidence of 16.9% (95% CI, 10.2-23.6) in current smokers versus 8.6% (95% CI, 7.0-10.2) in never smokers. Gray’s test confirmed a highly significant difference (*p* < .001). The Fine and Gray analysis reinforced this finding, showing that current smokers had more than double the risk of hip fractures compared to never smokers (SHR = 2.14; 95% CI, 1.34-3.42; *p* < .001).

For any fracture, the cumulative incidence at nine years was 42.8% (95% CI, 34.3-51.3) among current smokers compared to 36.6% (95% CI, 33.9-39.4) among never smokers. While Gray’s test approached significance (*p* = .098), the Fine and Gray model identified a significantly elevated risk for current smokers (SHR = 1.36; 95% CI, 1.05-1.78; *p* = .022).

Former smokers exhibited modest and generally non-significant increases in fracture risk compared to never smokers across all outcomes. Specifically, the SHR for hip fractures was 1.05 (95% CI, 0.81-1.38; *p* = .71), for MOF was 1.13 (95% CI, 0.98-1.31; *p* = .09), and for any fracture was 1.16 (95% CI, 1.02-1.31; *p* = .02). Only the SHR for the any fracture category achieved statistical significance (HR, 1.16; 95% CI, 1.02-1.31, *p* = .02; Table S5).

## Discussion

This study reaffirms current smoking as a potent risk factor for osteoporotic fractures, particularly hip fractures, in line with the established literature.^22^^,^^39^ Importantly, our findings illuminate the influence of smoking cessation, suggesting that fracture risk may gradually diminish with extended periods of abstinence. While the hazard ratios for ex-smokers did not consistently reach statistical significance below unity, the downward trend over time aligns with previous work indicating partial reversibility of smoking-induced skeletal damage.^40^ This observation underscores a valuable public health implication: promoting cessation programs could not only reduce tobacco-related comorbidities but also contribute to attenuating fracture risk in older adults. This work extends previous research by integrating smoking intensity and time since cessation with HR-pQCT-derived microarchitecture data and objective functional performance in joint mediation models, applied to radiographically verified fractures and explicitly accounting for competing mortality.

Our mediation analysis demonstrated that diminished physical function, exemplified by slower walking speed, emerged as a critical mediator linking smoking to heightened fracture risk in our cohort, complementing prior work that identified muscle weakness and functional decline as key contributors to falls and fractures.^41–43^ Although smokers exhibited subtle reductions in cortical and trabecular bone microarchitecture, these alterations alone did not account for the sizable elevation in fracture incidence, reinforcing the concept that smoking-related skeletal vulnerability extends beyond adversely affected bone health. Rather, frailty, sarcopenia, and impaired physical function, appear integral to translating smoking exposure into fracture events. In line with this multifactorial etiology, our mediation models suggest that HR-pQCT-derived total vBMD also contributes, albeit less prominently, to the indirect effect of smoking on fracture risk. Notably, while FN BMD showed some evidence of mediation in selected models, the lack of consistently significant results underscores that its role may be more modest within this sample. In sensitivity mediation analyses using alternative functional measures, TUG showed small but statistically significant indirect effects for any and hip fractures, whereas 30-s Chair Stand test was directionally consistent but not significant; both effects were smaller than those observed for walking speed and total vBMD. Consistent with this, adjustment for falls and OLS modestly weakened the smoking-fracture associations, which is consistent with a small contribution from balance and fall propensity. By contrast, adding calcium supplementation, osteoporosis medications, and estrogen therapy produced small increases in fracture hazard ratios, a pattern more compatible with confounding by indication than with attenuation. Some null or borderline findings for MOF and Chair-Stand test may reflect insufficient statistical power given the small number of current smokers and events, rather than a true absence of effect.

Taken together, these findings highlight that interventions aimed at improving physical function may be particularly potent in mitigating fracture risk among smokers, and they further imply a possible secondary avenue of risk attenuation via total vBMD. Future studies should seek to clarify whether these mediators reflect true mechanistic pathways or broader processes associated with smoking and fracture susceptibility, with an eye toward refining targeted preventive strategies. Additional pathways, such as vascular and neurocognitive mechanisms that impair postural control, merit investigation in future work.

In addition, emerging evidence points to novel mediators, such as cadmium exposure (a heavy metal prevalent in cigarette smoke), which has been directly linked to decreased bone density and increased fracture risk, particularly in Swedish populations.^44^^,^^45^ Hormonal dysregulation, notably the accelerated decline in estrogen in female smokers, has been identified as a critical pathway exacerbating bone loss.^46^ These findings highlight that smoking-induced skeletal vulnerability is multifactorial, encompassing both direct effects on bone cells and indirect effects through systemic mediators.

The competing risk analysis illustrating that smokers not only exhibit higher fracture incidence but also face increased mortality—a finding particularly salient in older populations with multiple comorbidities. The elevated mortality rate in current smokers may attenuate the apparent fracture burden if a simple Cox model is used without accounting for death as a competing event. However, the Fine and Gray subdistribution models confirmed a persistent, and in some cases amplified, fracture risk among current smokers even in the face of higher mortality. This result aligns with the biological plausibility that smoking accelerates systemic decline, thereby elevating both fracture and death risks.

In the sensitivity analysis, time since smoking cessation emerged as a critical determinant of skeletal health and survival, with ex-smokers demonstrating progressively lower fracture rates and reduced mortality risk as the duration of abstinence increased. Each additional year of abstinence was associated with ~1% lower risk of any and hip fracture (HR per year 0.99, 95% CI, 0.98-1.00) and with reduced mortality while the MOF association was not significant.

Specifically, cessation for five to ten years significantly decreased the risk of any fracture and hip fracture, while cessation for 15 or more years provided marked protection against mortality. Notably, studies have also shown that improvements in bone turnover markers and recovery of microarchitectural integrity correlate with the length of time since cessation, suggesting that some of the deleterious effects of smoking on bone may be reversible over a sustained period.^15^ These findings suggest that the benefits of smoking cessation on bone health and overall survival accrue progressively over time, potentially through mechanisms such as improved bone turnover, stabilization of bone architecture, and enhanced functional capacity.

In contrast, individuals classified as moderate and heavy smokers exhibited significantly elevated risks of hip fractures and mortality compared to never-smokers, underscoring the detrimental impact of sustained heavy smoking on musculoskeletal integrity and longevity. Although low-level smoking did not result in statistically significant increases in these risks, there were observable trends toward higher hazards, and the lack of statistical significance could be due to low statistical power in the analyses. Additionally, the role of other potential mediators, including cadmium exposure and sex hormone dysregulation, cannot be excluded and warrants further investigation. Collectively, these results highlight the substantial long-term benefits of smoking cessation, and the persistent risks associated with continued moderate to heavy smoking, emphasizing the importance of sustained abstinence for improving skeletal health and reducing mortality.

This investigation offers several strengths. The large sample size and long follow-up provided robust statistical power with post hoc calculations showing power exceeding 80% for detecting differences in many DXA and HR-pQCT derived bone traits and in the proportion of incident hip fractures between smokers and non-smokers. However, the power was insufficient for analyses of any fracture because the between-group differences were smaller. Another strength was the radiographic verification of all fractures, which reduces outcome misclassification bias. Our comprehensive study participant assessments, which encompasses bone parameters, biochemical markers, comorbidities, medications, and physical function, allow a multifaceted exploration of how smoking status might affect fracture risk. Moreover, the incorporation of a mediation analysis permits a nuanced understanding of the pathways linking smoking to fractures. Such insights provide actionable knowledge for clinicians and policymakers who aim to devise targeted interventions that integrate both smoking cessation efforts and mobility enhancement programs.

Notwithstanding these strengths, several limitations must be acknowledged. First, the number of current smokers was relatively small, compared to those of ex-smokers and never smokers, which may limit the precision of our estimates for ongoing tobacco users, especially in the mediation and intensity-stratified analyses.

Second, the observational design precludes definitive causal inferences, and residual confounding, such as differences in diet or socioeconomic factors, cannot be entirely ruled out. Third, smoking was assessed once at baseline and was not updated with time; thus, reverse causation cannot be excluded. Nevertheless, the observed risk ordering (current > former ≈ never) and the decline with longer cessation are not consistent with a “sick-quitter” pattern that would inflate risk among former smokers. Fourth, balance was captured by self-reported falls and OLS rather than instrumented measures, and prospective fall surveillance was not available. Additionally, the cohort comprised older women, restricting the generalizability to men or younger populations. Finally, self-reported smoking intensity/duration may be misclassified, likely biasing associations toward the null.

From a clinical and public health standpoint, these results underscore the critical importance of smoking cessation as part of comprehensive fracture prevention strategies, particularly in populations with established smoking histories. Even partial reversals of smoking-induced skeletal and functional impairments can confer meaningful benefits. In addition, addressing the mechanical contributors to fracture, including reduced walking speed and fall propensity appears essential for mitigating smoking-related fragility fractures. In practice, coupling cessation support with targeted mobility and balance programs may offer incremental protection against fractures. Future investigations should incorporate more underexamined mediators such as cadmium and hormonal fluctuation. Ultimately, the evidence presented here offers a clear impetus for integrating smoking cessation programs with functional rehabilitation in older adults to reduce both fracture incidence and the concurrent elevation in mortality risk.

## Supplementary Material

JBMR_Smoking_Fracture_Zoulakis_Supplementalv3_zjag028

## Data Availability

Data cannot be made publicly available for ethical and legal reasons. Such information is subject to legal restrictions according to national legislation. Specifically, in Sweden, confidentiality regarding personal information in studies is regulated in the Public Access to Information and Secrecy Act (SFS 2009:400). The data underlying the results of this study might be made available upon request, after an assessment of confidentiality. There is thus a possibility to apply to get access to certain public documents that an authority holds. In this case, the University of Gothenburg is the specific authority that is responsible for the integrity of the documents with research data. Questions regarding such issues can be directed to the head of the Institute of Medicine, Sahlgrenska Academy, University of Gothenburg, Gothenburg, Sweden. Contact information can be obtained from medicin@gu.se.
