# Supplementary material for: Impact of current and previous smoking on fracture risk in older women: the role of physical function, bone density, and bone microarchitecture
Source: J Bone Miner Res. 2026 Feb 10;41(10):1096–108. doi: 10.1093/jbmr/zjag028 (PMC13616106; doi:10.1093/jbmr/zjag028)
Supplement: Supplementary file 1 [file jbmr_smoking_fracture_zoulakis_supplementalv3_zjag028.docx]

**Supplementary Materials**

## **Impact of Smoking on Fracture Risk in Older Women: The Role of Physical Function, Bone Density and Bone Microarchitecture**

M. Zoulakis^1, 2^, M. Ambjörn^1^, R. Jaiswal^1^, K. F. Axelsson^1, 3^, H. Litsne^1^, L. Johansson^1, 4^, M. Lorentzon^1, 2^

^1^Sahlgrenska Osteoporosis Centre, University of Gothenburg, Department of Internal Medicine and Clinical Nutrition, Gothenburg, Sweden

^2^Department of Internal Medicine, Emergency Medicine and Geriatric Medicine, Sahlgrenska University Hospital, Mölndal, Sweden

^3^Region Västra Götaland, Närhälsan Norrmalm, Health Centre, Skövde, Sweden

^4^Department of Orthopedics, Sahlgrenska University Hospital, Mölndal, Sweden

Table of Contents

- Supplemental Table 1: Hazard ratios for fracture and mortality by smoking cessation duration - page 2
- Supplemental Table 2: Hazard ratios for fracure and mortality by smoking exposure - page 3
- Supplemental Table 3: Mediation analysis - page 4
- Supplemental Table 4: Annual cumulative incidence of fractures for smokers Aalen-Johansen - page 5
- Supplemental Table 5: Association of smoking duration with mortality rates - page 6
- Supplemental Table 6: Number of individuals with avalaible data – page 7
- Supplemental Figure 1: The cumulative fracture incidence Aalen-Johansen - page 8

**Supplemental Table 1. Hazard Ratios for Fracture and Mortality by Smoking Cessation Duration**

| **Outcome** | **Years Since Cessation** | **Events** | **HR** | **95% CI (Lower - Upper)** | **p** |
| --- | --- | --- | --- | --- | --- |
| Any Fracture |  |  |  |  |  |
|  | 0-5 | 25 | 1.21 | 0.72 - 2.02 | 0.48 |
|  | 5-10 | 22 | 0.52 | 0.30 - 0.89 | 0.02 |
|  | 10-15 | 43 | 0.91 | 0.59 - 1.39 | 0.65 |
|  | 15+ | 345 | 0.82 | 0.62 - 1.08 | 0.16 |
| Hip Fracture |  |  |  |  |  |
|  | 0-5 | 5 | 0.42 | 0.14 - 1.20 | 0.11 |
|  | 5-10 | 5 | 0.29 | 0.09 - 0.90 | 0.03 |
|  | 10-15 | 7 | 0.45 | 0.18 - 1.12 | 0.09 |
|  | 15+ | 67 | 0.41 | 0.25 - 0.66 | <0.001 |
| MOF |  |  |  |  |  |
|  | 0-5 | 17 | 1.00 | 0.55 - 1.84 | 0.99 |
|  | 5-10 | 17 | 0.55 | 0.29 - 1.04 | 0.07 |
|  | 10-15 | 31 | 0.93 | 0.57 - 1.53 | 0.79 |
|  | 15+ | 258 | 0.83 | 0.60 - 1.15 | 0.27 |
| Mortality |  |  |  |  |  |
|  | 0-5 | 10 | 0.54 | 0.27 - 1.08 | 0.08 |
|  | 5-10 | 17 | 0.56 | 0.31 – 1.03 | 0.06 |
|  | 10-15 | 29 | 0.68 | 0.42 – 1.11 | 0.12 |
|  | 15+ | 188 | 0.51 | 0.37 – 0.72 | <0.001 |

**Supplemental Table 1:** Hazard Ratios (HRs) and 95% confidence intervals (CIs) were estimated using Cox proportional hazards models, adjusted for average daily cigarette consumption, age, BMI, clinical risk factors and femoral neck BMD with current smokers as the reference group.

**Supplemental Table 2. Hazard Ratios for Fracture and Mortality by Smoking Exposure**

| **Outcome** | **Category** | **Events (N)** | **HR** | **95% CI (Lower - Upper)** | **P** |
| --- | --- | --- | --- | --- | --- |
| Any Fracture | Never-Smoker | 516 | 1 | 1 (Reference) |  |
| Any Fracture | Low (1-10) | 41 | 1.30 | 0.93 - 1.81 | 0.12 |
| Any Fracture | Moderate & Heavy (11+) | 22 | 1.43 | 0.93 - 2.20 | 0.10 |
| Hip Fracture | Never-Smoker | 114 | 1 | 1 (Reference) |  |
| Hip Fracture | Low (1-10) | 11 | 1.35 | 0.70 - 2.61 | 0.38 |
| Hip Fracture | Moderate & Heavy (11+) | 13 | 4.00 | 2.23 - 7.18 | <0.001 |
| MOF | Never-Smoker | 388 | 1 | 1 (Reference) |  |
| MOF | Low (1-10) | 29 | 1.20 | 0.81 - 1.78 | 0.37 |
| MOF | Moderate & Heavy (11+) | 18 | 1.57 | 0.97 - 2.52 | 0.06 |
| Mortality | Never-Smoker | 235 | 1 | 1 (Reference) |  |
| Mortality | Low (1-10) | 39 | 3.08 | 2.18 - 4.36 | <0.001 |
| Mortality | Moderate & Heavy (11+) | 17 | 2.43 | 1.48 - 3.99 | <0.001 |

**Supplemental Table 2**: Hazard Ratios (HRs) and 95% confidence intervals (CIs) were estimated using Cox proportional hazards models, adjusted for age, BMI, clinical risk factors and femoral neck BMD. P-values < 0.05 indicate statistical significance.

### **Supplemental Table 3a.** Mediation Analysis

| **Outcome** | **Effect** | **Walking Speed** | **p** | **Total vBMD** | **p** | **Femoral Neck BMD** | **p** |
| --- | --- | --- | --- | --- | --- | --- | --- |
| **Any Fracture** |  |  |  |  |  |  |  |
|  | **Indirect Effect (ACME)** | 0.004 (0.002, 0.010) | <0.001 | 0.004 (0.001, 0.010) | 0.002 | 0.002 (−0.001, 0.000) | 0.24 |
|  | **Direct Effect (ADE)** | 0.015 (−0.002, 0.030) | 0.07 | 0.016 (−0.001, 0.030) | 0.006 | 0.019 (0.0002, 0.040) | 0.05 |
|  | **Proportion Mediated** | 0.207 (0.062, 0.910) | 0.03 | 0.182 (0.040, 0.900) | 0.022 | 0.082 (−0.118, 0.510) | 0.25 |
|  | **Total Effect** | 0.019 (0.002, 0.040) | 0.03 | 0.020 (0.003, 0.040) | 0.020 | 0.021 (0.002, 0.040) | 0.03 |
|  | **Sobel Test** | z = 3.53 | < 0.001 | z = 2.76 | 0.006 | z =1.16 | 0.25 |
| **MOF** |  |  |  |  |  |  |  |
|  | **Indirect Effect (ACME)** | 0.003 (0.001, 0.010) | 0.004 | 0.003 (0.001, 0.010) | 0.002 | 0.002 (−0.001, 0.010) | 0.89 |
|  | **Direct Effect (ADE)** | 0.011(−0.005, 0.030) | 0.19 | 0.011 (−0.005, 0.030) | 0.19 | 0.012 (−0.004, 0.030) | 0.21 |
|  | **Proportion Mediated** | 0.223 (−1.37, 2.27) | 0.096 | 0.234 (−1.218, 1.700) | 0.09 | 0.016 (−0.312, 0.960) | 0.85 |
|  | **Total Effect** | 0.014 (−0.002, 0.030) | 0.096 | 0.014 (−0.002, 0.030) | 0.09 | 0.014 (−0.003, 0.030) | 0.21 |
|  | **Sobel Test** | z = 3.34 | < 0.001 | z =2.76 | 0.006 | z = 1.16 | 0.24 |
| **Hip Fracture** |  |  |  |  |  |  |  |
|  | **Indirect Effect (ACME)** | 0.0015 (0.0005, 0.003) | 0.002 | 0.0013 (0.0003, 0.003) | 0.002 | 0.0008 (−0.0006, 0.002) | 0.24 |
|  | **Direct Effect (ADE)** | 0.0002 (−0.008, 0.010) | 0.95 | 0.0013 (−0.009, 0.010) | 0.79 | 0.0013 (−0.009, 0.010) | 0.77 |
|  | **Proportion Mediated** | 0.859 (−4.83, 5.81) | 0.79 | 0.508 (−2.78, 3.12) | 0.61 | 0.363 (−2.05, 2.18) | 0.71 |
|  | **Total effect** | 0.0017 (−0.0072, 0.010) | 0.79 | 0.0026 (−0.0073, 0.010) | 0.61 | 0.0021 (−0.0080, 0.010) | 0.67 |
|  | **Sobel Test** | z = 3.12 | 0.002 | z =2.64 | 0.008 | z = 1.16 | 0.25 |

**Supplemental Table 3a.** Models included the exposure variable (smoking) and mediators: Walking Speed, total volumetric bone density (vBMD) and femoral neck BMD as centered variables, along with covariates such as age, BMI and clinical risk factors. Variance Inflation Factors (VIF) for all covariates were below 1.30 indicating no issues with multicollinearity. The mediation estimates for ACME = Average Causal Mediation Effect (indirect effect), ADE = Average Direct Effect, and Proportion Mediated were obtained through nonparametric bootstrap mediation with 1,000 simulations, while the Sobel test was used as an alternative method to assess the significance of the indirect effects. Estimates and 95% confidence intervals are rounded for clarity

**Supplemental Table 3b.** Sensitivity analysis mediation

| **Outcome** | **Effect** | **30-sec Chair stand** | **p** | **TUG** | **p** |
| --- | --- | --- | --- | --- | --- |
| **Any Fracture** |  |  |  |  |  |
|  | **Indirect Effect (ACME)** | 0.001(-0.0004, ~0.000) | 0.15 | 0.002(0.006, ~0.000) | 0.008 |
|  | **Direct Effect (ADE)** | 0.019(0.001 ,0.04) | 0.03 | 0.018(-0.000, 0.040) | 0.06 |
|  | **Proportion Mediated** | 0.058(-0.035, 0.350) | 0.17 | 0.110(0.013, 0.560) | 0.04 |
|  | **Total Effect** | 0.021(0.003, 0.040) | 0.02 | 0.021(0.002, 0040) | 0.03 |
|  | **Sobel Test** | z = 1.37 | 0.17 | z = 2.44 | 0.015 |
| **MOF** |  |  |  |  |  |
|  | **Indirect Effect (ACME)** | 0.001(-0.0003, ~0.000) | 0.15 | 0.002(0.004, ~0.000) | 0.008 |
|  | **Direct Effect (ADE)** | 0.014(-0.003 ,0.03) | 0.16 | 0.013(-0.004, 0.030) | 0.15 |
|  | **Proportion Mediated** | 0.071(-0.209, 0.730) | 0.20 | 0.107(-0.578, 0.960) | 0.11 |
|  | **Total Effect** | 0.015(-0.002, 0.030) | 0.08 | 0.021(-0.002, 0030) | 0.10 |
|  | **Sobel Test** | z = 1.36 | 0.17 | z = 2.325 | 0.02 |
| **Hip Fracture** |  |  |  |  |  |
|  | **Indirect Effect (ACME)** | 0.0005(-0.0001, ~0.000) | 0.15 | 0.0005(0.000,0.001) | 0.003 |
|  | **Direct Effect (ADE)** | 0.001(-0.008, 0010) | 0.89 | 0.002(-0.008, 0.010) | 0.74 |
|  | **Proportion Mediated** | 0.386(-1.59, 1.60) | 0.81 | 0.238(-1.34, 1.14) | 0.69 |
|  | **Total effect** | 0.001(-0.007, 0.010) | 0.81 | 0.002(-0.007, 0.010) | 0.69 |
|  | **Sobel Test** | z = 1.33 | 0.18 | z = 2.01 | 0.04 |

**Supplemental Table 3b.** Same analytic specifications as Supplemental Table 3a but replacing mediators with TUG (Timed Up and Go ) and 30-seconds Chair Stand test.

**Supplemental Table 4.** Annual cumulative incidence

| **Category** | **Total** | **Fracture Events** | **Death Events** | **1 Years** | **2 Years** | **3 Years** | **4 Years** | **5 Years** | **6 Years** | **7 Years** | **8 Years** | **9 Years** | **Gray’s** |
| --- | --- | --- | --- | --- | --- | --- | --- | --- | --- | --- | --- | --- | --- |
| **MOF** |  |  |  |  |  |  |  |  |  |  |  |  | 0.181 |
| Never Smokers | 1524 | 388 | 145 | 3.7%  (2.7–4.6) | 6.6%  (5.3–7.8) | 9.3% (7.9–10.8) | 13.1% (11.4–14.8) | 16.3% (14.4–18.1) | 18.8% (16.9–20.8) | 21.5% (19.5–23.6) | 24.0% (21.8–26.2) | 27.8 (25.2–30.3) |  |
| Smokers | 157 | 48 | 40 | 3.2% (0.4–5.9) | 5.7% (2.1–9.4) | 10.8% (6.0–15.7) | 15.3% (9.6–20.9) | 21.0% (14.6–27.4) | 22.3% (15.8–28.8) | 24.8% (18.1–31.6) | 27.7% (20.6–34.7) | 32.9% (24.6–41.1) |  |
| **Hip Fracture** |  |  |  |  |  |  |  |  |  |  |  |  | **< 0.001** |
| Never Smokers | 1524 | 114 | 208 | 0.5% (0.1–0.8) | 1.2% (0.6–1.7) | 1.8% (1.2–2.5) | 2.7% (1.9–3.5) | 3.6% (2.7–4.5) | 4.5% (3.5–5.6) | 5.4% (4.3–6.5) | 7.0% (5.6–8.3) | 8.6% (7.0–10.2) |  |
| Smokers | 157 | 24 | 48 | 0.0% (0.0–0.0) | 0.6% (0.0–1.9) | 2.5% (0.1–5.0) | 3.8% (0.8–6.8) | 7.6% (3.5–11.8) | 8.3% (4.0–12.6) | 11.5% (6.5–16.5) | 12.2% (7.0–17.3) | 16.9% (10.2–23.6) |  |
| **Any Fracture** |  |  |  |  |  |  |  |  |  |  |  |  | 0.098 |
| Never Smokers | 1524 | 516 | 127 | 5.4% (4.2–6.5) | 9.5% (8.0–11.0) | 13.2% (11.5–14.9) | 18.0% (16.1–20.0) | 21.9% (19.8–23.9) | 25.5% (23.3–27.7) | 29.0% (26.7–31.2) | 32.4% (30.0–34.8) | 36.6% (33.9–39.4) |  |
| Smokers | 157 | 64 | 33 | 5.7% (2.1–9.4) | 9.6% (4.9–14.2) | 15.9% (10.2–21.7) | 21.0% (14.6–27.4) | 28.0% (21.0–35.1) | 31.8% (24.5–39.2) | 35.0% (27.5–42.5) | 37.9% (30.2–45.5) | 42.8% (34.3–51.3) |  |

### **Supplemental Table 4.** Cumulative incidence estimates are provided at annual intervals with corresponding 95% confidence intervals in parentheses, calculated using the Aalen-Johansen estimator. Gray's test to compare the cumulative incidence functions between groups.

**Supplemental Table 5.** Fine and Gray Competing Risk Analysis

| **Smokers** | **Fracture** | **SHR** | **Lower CI** | **Upper**  **CI** | **p** |
| --- | --- | --- | --- | --- | --- |
|  |  |  |  |  |  |
|  | Hip | **2.14** | **1.34** | **3.42** | **<0.001** |
|  | MOF | 1.36 | 0.998 | 1.85 | 0.051 |
| **Former smokers** | Any | **1.36** | **1.05** | **1.78** | **0.022** |
|  |  |  |  |  |  |
|  | Hip | 1.05 | 0.805 | 1.38 | 0.71 |
|  | MOF | 1.13 | 0.982 | 1.31 | 0.09 |
|  | Any | **1.16** | **1.02** | **1.31** | **0.02** |

### **Supplemental Table 5.** SHR = Subdistribution hazard Ratio; CI = Confidence Interval. SHRs were estimated using Fine-Gray competing risk, accounting for death as a competing event. P-values indicate the statistical significance of each covariate. Smoking status was analyzed as a binary variable (0 = Never Smoker, 1 = Current Smoker or Former Smokers). The model was adjusted for age, body mass index (BMI), previous fractures, rheumatoid arthritis, steroid use, family history of fractures, alcohol intake, and femoral neck BMD. Statistically significant associations (p < 0.05) are emphasized.

**Supplemental table 6. Number of individuals with available data per group and variable**

| **Total number in cohort** | **Smokers** | **Former Smokers** | **Never smokers** |
| --- | --- | --- | --- |
|  |  |  |  |
| BMD Femoral Neck | 155 | 1335 | 1521 |
| BMF Total Hip | 155 | 1335 | 1521 |
| BMD Lumbar Spine | 152 | 1329 | 1516 |
| BMD Radius | 157 | 1332 | 1516 |
| Trabecular Bone Score | 152 | 1329 | 1516 |
| BMSi | 25 | 274 | 343 |
|  |  |  |  |
| **Ultra distal tibia microarchitecture** |  |  |  |
| Total area | 154 | 1292 | 1478 |
| Total vBMD | 154 | 1292 | 1478 |
| Cortical vBMD | 154 | 1291 | 1479 |
| Cortical area | 154 | 1924 | 1479 |
| Tr Th | 153 | 1292 | 1478 |
| Tr Sp | 149 | 1285 | 1477 |
| Tr BV/TV | 154 | 1292 | 1478 |
| Cortical perimeter | 154 | 1291 | 1479 |
| Cortical porosity | 154 | 1291 | 1476 |
| Stiffness | 154 | 1291 | 1478 |
| Failure load | 154 | 1291 | 1478 |
|  |  |  |  |
| **Ultra distal radius** **microarchitecture** |  |  |  |
| Total area | 127 | 1083 | 1232 |
| Total vBMD | 127 | 1083 | 1232 |
| Cortical vBMD | 127 | 1083 | 1233 |
| Cortical area | 127 | 1083 | 1233 |
| Tr Th | 127 | 1081 | 1231 |
| Tr Sp | 126 | 1078 | 1227 |
| Tr BV/TV | 127 | 1083 | 1227 |
| Cortical perimeter | 127 | 1081 | 1232 |
| Cortical porosity | 127 | 1082 | 1228 |
| Stiffness | 127 | 1083 | 1229 |
| Failure load | 127 | 1083 | 1229 |
|  |  |  |  |
| **Physical function and Sarcopenia** |  |  |  |
| Walking Speed | 153 | 1334 | 1541 |
| 30s Chair stand | 155 | 1328 | 1510 |
| Grip Strength | 150 | 1288 | 1475 |
| One leg stand | 114 | 1053 | 1239 |
| PASE | 157 | 1337 | 1519 |
| TUG | 154 | 1335 | 1511 |
| AWGS | 153 | 1330 | 1515 |
| EWGSOP2 | 156 | 1332 | 1513 |
| SDOC | 154 | 1330 | 1511 |

**Supplemental Figure 1.** Cumulative incidence of fractures: A (Any Fracture) , B MOF (Major Osteoporotic Fracture), C (Hip Fracture) over up to 9 years follow-up, stratified by smoking status. Shaded regions represent 95% confidence intervals. The cumulative incidence function was estimated using the Aalen-Johansen estimator, with death treated as a competing risk.
